# Halitosis and *helicobacter pylori* infection: A meta-analysis

**DOI:** 10.1097/MD.0000000000004223

**Published:** 2016-09-30

**Authors:** Wenhuan Dou, Juan Li, Liming Xu, Jianhong Zhu, Kewei Hu, Zhenyu Sui, Jianzong Wang, Lingling Xu, Shaofeng Wang, Guojian Yin

**Affiliations:** Department of Gastroenterology, The Second Affiliated Hospital of Soochow University, Suzhou, Jiangsu Province, People's Republic of China.

**Keywords:** halitosis, *Helicobacter pylori*, meta-analysis

## Abstract

**Background:**

Halitosis is used to describe any disagreeable odor of expired air regardless of its origin. Numerous trials published have investigated the relation between *Helicobacter pylori* (*H pylori*) infection and halitosis, and even some regimes of *H pylori* eradication have been prescribed to those patients with halitosis in the clinic. We conducted a meta-analysis to define the correlation between *H pylori* infection and halitosis.

**Objectives:**

To evaluate whether there is a real correlation between *H pylori* infection and halitosis, and whether *H pylori* eradication therapy will help relieve halitosis.

**Methods:**

We searched several electronic databases (The Cochrane Library, MEDLINE, EMBASE, PubMed, Web of Science, and Wanfangdata) up to December 2015. Studies published in English and Chinese were considered in this review. After a final set of studies was identified, the list of references reported in the included reports was reviewed to identify additional studies. Screening of titles and abstracts, data extraction and quality assessment was undertaken independently and in duplicate. All analyses were done using Review Manager 5.2 software.

**Results:**

A total of 115 articles were identified, 21 of which met the inclusion criteria and presented data that could be used in the analysis. The results showed that the OR of *H pylori* infection in the stomach between halitosis-positive patients and halitosis-negative patients was 4.03 (95% CI: 1.41–11.50; *P* = 0.009). The OR of halitosis between *H pylori*-positive patients and *H pylori*-negative patients was 2.85 (95% CI: 1.40–5.83; *P* = 0.004); The RR of halitosis after successful *H pylori* eradication in those *H pylori*-infected halitosis-positive patients was 0.17 (95% CI: 0.08–0.39; *P* <0.0001), compared with those patients without successful *H pylori* eradication. And the RR of halitosis before successful *H pylori* eradication therapy was 4.78 (95% CI: 1.45–15.80; *P* = 0.01), compared with after successful *H pylori* eradication therapy.

**Conclusions:**

There is clear evidence that *H pylori* infection correlates with halitosis. *H pylori* infection might be important in the pathophysiological mechanism of halitosis, and *H pylori* eradication therapy may be helpful in those patients with refractory halitosis.

## 1 Introduction

The term halitosis or bad breath is generally defined to describe any noticeable disagreeable odor of expired air regardless of its origin.^[1]^ The diagnosis of halitosis can always be genuine halitosis, pseudo halitosis, and halitophobia.^[2]^ As a public social health problem, genuine halitosis is always classified into physiological and pathophysiological illness, which affects a significant number of people around the world. Research reveals that nearly 50% of the adult population has halitosis.^[3]^

The cause of the halitosis is often considered to be found in the oral cavity. It was found that 80% to 90% of patients with halitosis was caused by oral conditions, defined as bad breath or oral malodor.^[4]^ Halitosis usually results from deep caries, pericoronitis, periodontal disease, exposed necrotic tooth pulps, peri-implant disease, imperfect dental restorations, unclean dentures, tongue coating, mucosal lesions, and factors causing decreased salivary flow rate.^[4]^ The causative organisms from halitogenic biofilm on the posterior dorsal tongue, and/or within gingival crevices/periodontal pockets are usually gram-negative anaerobic bacteria. The basic pathophysiological process is microbial degradation of sulfur containing amino acid substrates, for example, methionine, cysteine, and cysteine.^[5]^ Bacterial metabolism of these kinds of amino acids leads to metabolites including many compounds, such as volatile sulfur compounds (VSC), for example, hydrogen sulfide (H_2_S), methyl mercaptan (MM, CH_3_SH), dimethyl sulfide, and skatole, indole.^[6]^ The main odorants implicated in intraoral halitosis are MM and H_2_S.^[7]^

About 10% to 20% of all genuine halitosis cases are attributed to extra-oral diseases,^[5]^ including upper and lower respiratory tract disorders, gastrointestinal disorders, some systemic diseases, metabolic diseases, medications, and cancer.^[4]^ Some authorities have reported that the ears, nose, and throat (ENT) are the most common sites of origin of extra-oral halitosis,^[5]^ and it is well established that various ENT disorders and symptoms may be a manifestation of gastroesophageal reflux disease (GERD). Poelmans et al^[8]^ showed that patients with suspected GERD-related ENT symptoms had a high prevalence of esophagitis and this was associated with better response to antisecretory therapy. Moshkowitz et al^[9]^ found that halitosis was a frequent symptom of GERD and might be considered an extra-esophageal manifestation of GERD. Struch et al^[10]^ showed clear evidence for an association between GERD and halitosis and suggested treatment options for halitosis, such as proton pump inhibitors.

It was Tiomny et al^[11]^ who first showed the possible connection between halitosis and *Helicobacter pylori* (*H pylori*) infection in a case report. Since then, *H pylori* infection has been investigated with regards to a potential relationship with halitosis in the past 20 years in many studies, and inconsistent results from case reports, epidemiological studies, randomized controlled trials, and quasirandomized controlled trials have been reported.^[7,11–29]^ For example, data from Ierardi et al^[13]^ showed that *H pylori* eradication could resolve the symptom of halitosis. Serin et al^[19]^ showed that halitosis was a frequent and treatable symptom of *H pylori*-positive nonulcer dyspepsia and suggested an *H pylori* eradication therapy for those patients with halitosis. However, on the contrary, in Tangerman study no association between halitosis and *H pylori* infection was found and he concluded that halitosis always originated within the oral cavity and seldom or never within the stomach.^[7]^

In order to clarify the possible relation between the *H pylori* infection and the annoying halitosis, we conducted an exhaustive review and meta-analysis of all the literatures related to this subject to evaluate whether *H pylori* is a cause of halitosis and whether eradication of *H pylori* can relieve it.

## 2 Methods

### 2.1 Search strategy

The Medical Ethics Committee of a 3-A hospital, the second Affiliated Hospital of Suzhou University, Suzhou, China, approved the study. Due to the review nature of the study, informed consent was waived. A comprehensive, computerized literature search was conducted in MEDLINE, PubMed, Web of Science, and Wanfangdata from the beginning of indexing for each database to December 2015, by 2 independent investigators (GY and WD). Articles published in English and Chinese were considered in this review. Search terms included: “halitosis,” “bad breath,” or “malodor,” combined with *Helicobacter pylori*, or “urea breath test.” The title and abstract of eligible studies were then reviewed to exclude any study that was irrelevant to the research question. After a final set of studies was identified, the list of references reported in the included reports was reviewed to identify additional studies. We did not include data presented only as abstracts at conferences.

### 2.2 Study selection and data extraction

Two review authors, Guojian Yin and Wenhuan Dou independently assessed the abstracts of studies resulting from the searches. Studies were included if they met the following criteria: published as an original article; published as case reports, case-control studies, cross-sectional studies, randomised/quasi-randomised controlled trials, and comparative clinical experimental trial; the relation between the incidence of halitosis and *H pylori* infection, or the incidence of halitosis before and after eradication therapy of *H pylori*, were investigated in these articles. Full text copies of any relevant and potentially relevant studies, those appearing to meet the inclusion criteria, or for which there were insufficient data in the title and abstract to make a clear decision, were obtained. The full articles were assessed independently by 2 review authors and any disagreement on the eligibility of included studies was resolved through discussion and consensus.

### 2.3 Statistical analysis

All studies were grouped and analyzed on the basis of study design: *H pylori* infection rates in patients with or without halitosis (group 1); Halitosis rates in patients with or without *H pylori* infection (group 2); Halitosis rates in infected halitosis patients after the treatment with or without successful *H pylori* eradication (group 3); Halitosis rates in *H pylori*-infected patients before and after successful *H pylori* eradication (group 4).

The Cochrane Q-statistic and the *I*^2^-statistic were used to assess statistical heterogeneity between studies, and an *I*^2^ value of >50% or a *P* value <0.05 for the Q-statistic was taken to suggest significant heterogeneity.^[30]^ In the presence of heterogeneity, the random-effects model is recommended by the Cochrane collaboration, because its assumptions account for the presence of variability among studies.^[31,32]^ As the included studies in each subgroup were less than 10, the publication bias was not assessed through Funnel plot or Begg test^[33]^ and Egger tests in this study.^[34]^

All statistical tests were 2-tailed, and a probability level of *P* <0.05 was considered significant. Results were presented in accordance with the guidelines proposed by MOOSE.^[35]^ ORs (case-control studies, cross-sectional studies) and RRs (randomized controlled trials and quasi-randomized controlled trials, or comparative clinical experimental trials) were used as the reporting different risk estimates. All analyses were done using Review Manager 5.2 software.

## 3 Results

**Figure 1:**
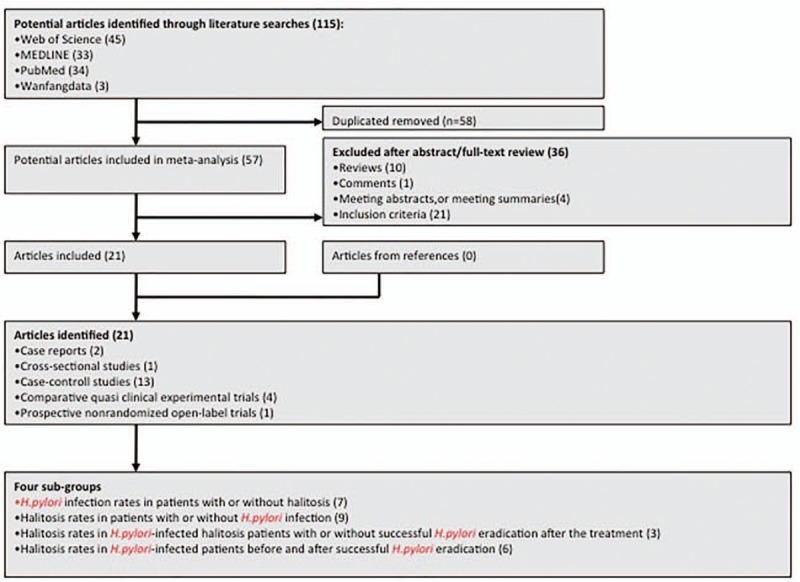
The flow sheet of the studies and the corresponding classification by study design.

The search strategy generated 115 citations, of which 57 were considered of potential value. Thirty-six of these 57 articles were subsequently excluded from the meta-analysis for various reasons (21 studies were excluded by inclusion criteria, 10 reviews, 4 meeting abstracts/summaries, and 1 comment). No additional article was included from the reference of the included 21 articles even after an overall and careful inspection of those references. In the final analysis 21 articles (2 case reports, 1 cross-sectional study, 13 case-control studies, 4 comparative quasi clinical experimental trials, and 1 prospective nonrandomized open-label trial) were included. Figure 1 shows the flow-sheet of these studies and their classification by study design. Four subgroups were further classified in the final meta-analysis.

The characteristics of the studies are shown in Table 1. The publication dates of the studies included in the meta-analysis ranged from 1992 to 2015. The 21 studies were ranked as moderate quality. A total of 5062 participants were involved in these studies (2312 participants in group 1, 2052 participants in group 2, 128 participants in group 3, and 570 participants in group 4).

**Table 1:**
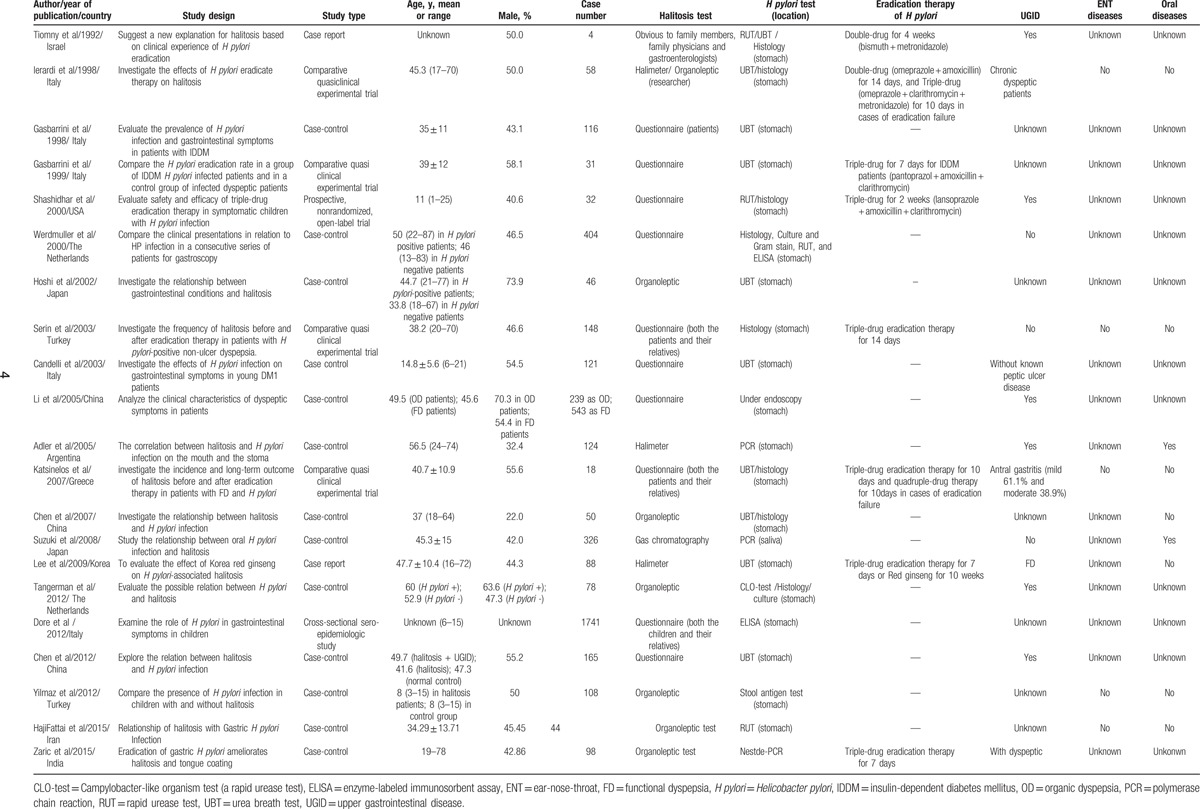
The characteristics of the included studies in this meta-analysis.

### 3.1 Group 1 (n = 7): *H pylori* infection rates in patients with or without halitosis

**Figure 2:**
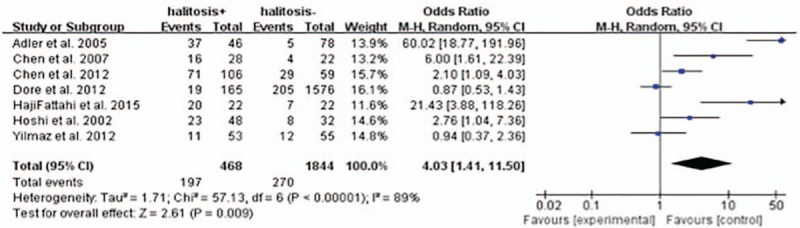
*H pylori* infection rates in patients with or without halitosis.

Halitosis rates were associated with a statistically significant increase of *H pylori* infection as shown by the random-effects model: Overall OR is 4.03 (1.41–11.50) with *P* <0.05 (Fig. 2). Evidence of heterogeneity was shown between the studies: The *I*^2^ was 89 and *P* <0.05. The random-effects meta-analysis was therefore applied to minimize the effects of heterogeneity.

### 3.2 Group 2 (n = 9): halitosis rates in patients with or without *H pylori* infection

**Figure 3:**
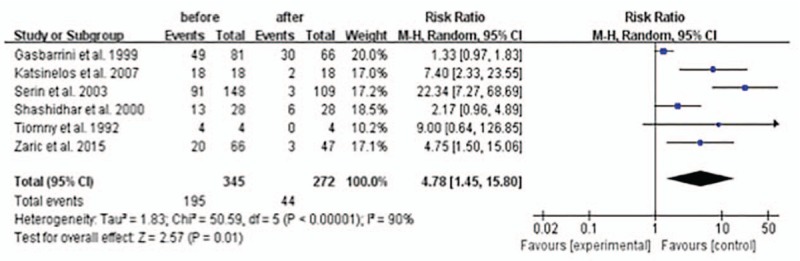
Halitosis rates in patients with or without *H pylori* infection.

*H pylori* infection rate were associated with a statistically significant increase of halitosis as shown by the random-effects model: overall OR is 2.85 (1.40–5.83) with *P* <0.01 (Fig. 3). Evidence of heterogeneity was shown between the studies: The *I*^2^ was 87.20 and *P* <0.05. The random-effects meta-analysis was therefore used to minimize the effects of heterogeneity.

### 3.3 Group 3 (n = 3): halitosis rates in *H pylori*-infected halitosis patients after the treatment with or without successful *H pylori* eradication

**Figure 4:**
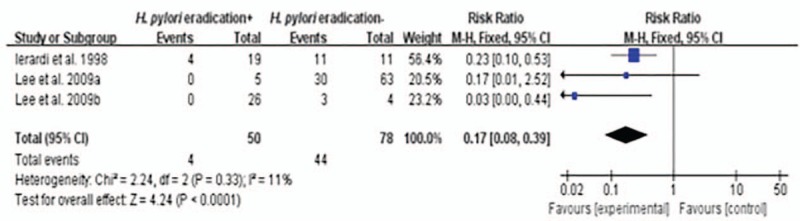
Halitosis rates in *H pylori*-infected patients with halitosis after treatment with or without successful *H pylori* eradication.

Compared with the halitosis rates of those *H pylori*-infected halitosis patients without successful *H pylori* eradication after the treatment, the halitosis rates of the patients with successful *H pylori* eradication were lower with a statistical significance: overall RR is 0.17 (0.08–0.39) with *P* <0.0001 (Fig. 4). There was no evidence of heterogeneity between the studies (the *I*^2^ was 11.00 and *P* >0.05). The fixed-effects meta-analysis was therefore chosen to assess the overall RR effects.

### 3.4 Group 4 (n = 5): halitosis rates in *H pylori*-infected patients before and after successful *H pylori* eradication

**Figure 5:**
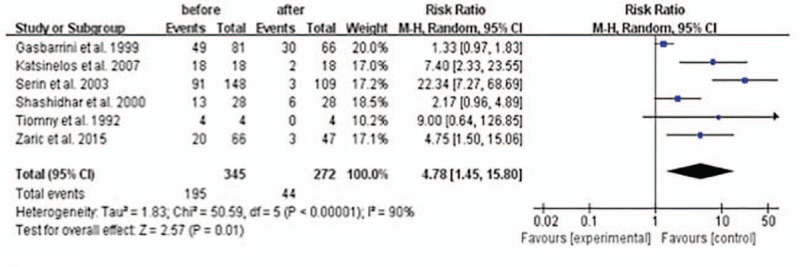
Halitosis rates in *H pylori*-infected patients before and after successful *H pylori* eradication.

Compared with the halitosis rates in those *H pylori*-infected patients before the successful *H pylori* eradication, it was lower after successful *H pylori* eradication therapy with a statistical significance: overall RR is 4.78 (1.45–15.80) with *P* <0.05 (Fig. 5). There was evidence of heterogeneity between the studies: the *I*^2^ was 90 and *P* <0.05 suggesting evidence of heterogeneity. The random-effects meta-analysis was therefore chosen to minimize the effects of heterogeneity.

## 4 Discussion

The exact pathophysiological mechanism of halitosis is not clear. The possible relationship between *H pylori* infection and halitosis was first suggested by Marshall et al^[36]^ in 1985. The potential relation between halitosis and *H pylori* infection was further found by Tiomny et al^[11]^ through a study of the effects of *H pylori* eradication therapy on halitosis. Since then, a lot of studies have been focused on this controversial subject.

In 2002, Hoshi et al^[17]^ proved that the intensity of malodor of mouth air was higher in *H pylori*-positive patients than in *H pylori*-negative patients, and the levels of H_2_S and dimethyl sulfide in mouth air were also significantly higher in the *H pylori*-positive patients than in the *H pylori*-negative patients. In 2005, Adler et al^[37]^ showed that the detection of *H pylori* by histopathology in the gastric biopsies was positive in 80.43% patients with halitosis and only 6.41% patients without halitosis (*P* <0.01). However, all these studies were carried out in the adults, the outcomes of the studies in the children were on the contrary. No statistical significance was reached between halitosis-positive children and halitosis-negative children by Dore et al^[25]^ and Yilmaz et al.^[27]^

In this meta-analysis, we took all these studies into account without considering the differences of age or sex between them. The results showed that the OR (random model) of *H pylori* infection between halitosis-positive patients and halitosis-negative patients was 4.03 (95% CI: 1.41–11.50; *P* = 0.009). We also did the meta-analysis of the risk of halitosis in the *H pylori*-positive patients versus *H pylori*-negative patients. The results showed that the OR (random model) of halitosis between *H pylori*-positive patients and *H pylori*-negative patients was 2.85 (95% CI: 1.40–5.83; *P* = 0.004).

But how does *H pylori* infection produce halitosis. By assessing the VSC produced by 3 strains of *H pylori* (ATCC 43504, SS 1, and DSM 4867) in broth cultures mixed with different sulfur-containing amino acids in vitro, Lee et al^[38]^ showed that *H pylori* was capable of producing H_2_S and MM. Although the production of VSC by *H pylori* was a little bit different among different strains of *H pylori* and different sulfur-containing amino acids, it was still the direct evidence that this microorganism can contribute to the development of halitosis. It was suggested that the VSC produced in the gastrointestinal tract could diffuse into the lung air after being carried to the lungs via blood.^[17,39]^ Yoo et al^[40]^ found that erosive changes in esophagogastroduodenal mucosa were strongly correlated with increased VSC levels, suggesting that *H pylori*-associated eroded and inflamed mucosa might aggravate halitosis by making VSC diffusion much easier into blood. It was also shown that in accordance with higher levels of VSC produced in patients with erosive mucosal changes and ulcerative changes, the enzymes cystathionine β-synthase (CBS) and cystathionine γ-lyase (CSE) prerequisite for VSC generation were obviously highly induced.^[40]^ On the contrary, Hoshi et al^[17]^ found that although levels of H_2_S and dimethyl sulfide in mouth air were significantly higher in *H pylori*-positive patients than in *H pylori*-negative patients, which meant *H pylori* did have some relation with halitosis, but no significant difference was observed in the exhaled breaths between the 2 groups, which indicated the higher production of VSC in upper gastrointestinal tract might be not the main source of halitosis. Although the role of *H pylori* infection in the pathophysiological mechanism of halitosis and the increase of VSC level is not clear, it may still be a frequent contributor to the production of halitosis.

The oral HP infection has also been under investigation in the past. In1989, *H pylori* was found in dental plaque by bacterial culture.^[41]^ In 1991, Desai et al^[42]^ showed that *H pylori* was detected in dental plaque and in gastric antral and body mucosa in 98%, 67%, and 70%, respectively, of 43 consecutive patients with dyspepsia. In 1996 in a group of 100 dyspeptic subjects, *H pylori* was detected by campylobacter-like organism test (CLO-test) in saliva, dental plaques, and gingival pockets in 84%, 100%, and 100% of cases and by the culture in 55%, 88%, and 100%, respectively. The presence of *H pylori* determined by urea breath test (UBT) in the stomach in these subjects was 60%.^[43]^ Whether the *H pylori* infection in the oral cavity correlates with halitosis is not clear, further detailed investigation is needed.

In this meta-analysis we also evaluated the effect of *H pylori* eradication therapy on halitosis. The results showed that the RR (fixed model) of halitosis after successful *H pylori* eradication in the stomach in those *H pylori*-infected halitosis-positive patients was 0.17 (95% CI: 0.08–0.39; *P* <0.0001), compared with those patients without successful *H pylori* eradication. We also did the meta-analysis of the halitosis rates in *H pylori*-infected patients before and after successful *H pylori* eradication therapy. The results showed that the RR (random model) of halitosis before successful *H pylori* eradication therapy was 4.78 (95% CI: 1.45–15.80; *P* = 0.01), compared with the patients after successful *H pylori* eradication therapy. These results all favor successful *H pylori* eradication therapy to treat those patients of halitosis.

Interestingly, in 2011, Shalchi et al^[26]^ took an further comparative quasiexperimental clinical trial study of 33 halitosis-positive patients without oral diseases (17 *H pylori*-positive patients and 16 *H pylori*-negative patients). All patients received 2-week's *H pylori* eradication therapy regardless of *H pylori* infection. She found that the RRs of halitosis resolution in *H pylori*-positive group over *H pylori*-negative group were 2.8 and 3.3 respectively, and *H pylori* eradication could resolve halitosis in a majority of patients. It was suggested that *H pylori* might be a probable rather than a possible cause of halitosis.

In conclusion, there was a clear correlation between *H pylori* infection and halitosis. *H pylori* might be a common contributor to the production of halitosis. In those refractory halitosis-positive patients without any oral/ENT/systemic diseases, *H pylori* eradication therapy in the clinic may be helpful.
